# Coronavirus Pandemic and Worries during Pregnancy; a Letter to Editor

**Published:** 2020-03-16

**Authors:** Farzaneh Rashidi Fakari, Masoumeh Simbar

**Affiliations:** 1 Student Research Committee, Department of Midwifery and Reproductive Health, School of Nursing and Midwifery, Shahid Beheshti University of Medical Sciences, Tehran, Iran.; 2 Midwifery and Reproductive Health Research Center, Shahid Beheshti University of Medical Sciences, Tehran, Iran.

**Keywords:** Anxiety, Birth, COVID-19, pregnancy, infant, newborn

**Dear editor:** Coronavirus (COVID-19) is a new respiratory disease that is spreading widely throughout the world(1). There is no valid information available on pregnant women and their complications. But given previous epidemics (SARS and MERS), as well as mental and physical changes during pregnancy (2), pregnant women are more likely to be affected by the virus.

On the other hand, the Coronavirus epidemic has created stress and anxiety for pregnant women in different parts of the world. Concern and stress in pregnancy are associated with side effects such as preeclampsia, depression, increased nausea and vomiting during pregnancy, preterm labor, low birth weight, and low APGAR score (3-7). 

In the Coronavirus pandemic, pregnant women cited the following reasons for their concerns:

- Many pregnant women have had a birth plan before the pandemic, but are currently worried about how their families (mothers) will be present, given the urban and quarantine constraints; moreover, even if there is no inter-urban restriction, they may be worried about their families being infected during transportation.

- Many pregnant women do not go to visit their physicians due to concerns that they may be exposed to the Coronavirus in the hospital environment or on the way to the hospital and may be post-term. Or on the contrary, due to stress and worry they want an early termination and elective cesarean section.

- Many pregnant mothers are employed and constantly use sodium hypochlorite and alcohol detergents to control and prevent the virus, which can lead to poisoning. Some other pregnant mothers might become highly stressed and anxious, and overuse these detergents.

- Some mothers are worried about their fetal or their neonate being born. Also, some mothers worry about postpartum such as breastfeeding, and neonatal care (postpartum vaccination, screening).

Increasing mothers' awareness about the transmission of Coronavirus, risk factors, and red flags, as well as providing tele-counseling for pregnancy care and tele-triage could help reduce their anxiety and worry. It is also recommended that in cities where home birth and home services after birth are available, the medical team provide these services at home while maintaining safety.
